# Impact of Plasma Oxidative Stress Markers on Post-race Recovery in Ultramarathon Runners: A Sex and Age Perspective Overview

**DOI:** 10.3390/antiox10030355

**Published:** 2021-02-27

**Authors:** Carlos Guerrero, Eladio Collado-Boira, Ignacio Martinez-Navarro, Barbara Hernando, Carlos Hernando, Pablo Balino, María Muriach

**Affiliations:** 1 Department of Medicine, Jaume I University, 12001 Castellon, Spain; cguerrer@uji.es (C.G.); hernandb@uji.es (B.H.); balino@uji.es (P.B.); 2 Faculty of Health Sciences, Jaume I University, 12001 Castellon, Spain; colladoe@uji.es; 3 Department of Physical Education and Sport, University of Valencia, 46010 Valencia, Spain; ignacio.martinez-navarro@uv.es; 4 Sports Health Unit, Vithas-Nisa 9 de Octubre Hospital, 46001 Valencia, Spain; 5 Sport Service, Jaume I University, 12001 Castellon, Spain; hernando@uji.es; 6 Department of Education and Specific Didactics, Jaume I University, 12001 Castellon, Spain

**Keywords:** ultraendurance exercise, oxidative stress, antioxidants, muscle injury

## Abstract

Oxidative stress has been widely studied in association to ultra-endurance sports. Although it is clearly demonstrated the increase in reactive oxygen species and free radicals after these extreme endurance exercises, the effects on the antioxidant defenses and the oxidative damage to macromolecules, remain to be fully clarified. Therefore, the aim of this study was to elucidate the impact of an ultramarathon race on the plasma markers of oxidative stress of 32 runners and their post-race recovery, with especial focused on sex and age effect. For this purpose, the antioxidant enzymes glutathione peroxidase (GPx) and glutathione reductase (GR) activity, as well as the lipid peroxidation product malondialdehyde (MDA) and the carbonyl groups (CG) content were measured before the race, in the finish line and 24 and 48 h after the race. We have reported an increase of the oxidative damage to lipids and proteins (MDA and CG) after the race and 48 h later. Moreover, there was an increase of the GR activity after the race. No changes were observed in runners’ plasma GPx activity throughout the study. Finally, we have observed sex and age differences regarding damage to macromolecules, but no differences were found regarding the antioxidant enzymes measured. Our results suggest that several basal plasma markers of oxidative stress might be related to the extent of muscle damage after an ultraendurance race and also might affect the muscle strength evolution.

## 1. Introduction

Ultramarathon races are defined as sport events that involve running and/or walking distances greater than the 42,195 km of a marathon. In the recent years, these short of competitive events have gained a lot of popularity. These extremely long races defiance our physiological systems inducing muscle injuries (muscle membrane disruption), respiratory fatigue, cardiac and renal damage, representing an outstanding model to evaluate the ultra-endurance exercises/sports on human body physiology [1,2,3,4].

Oxidative stress is defined as the imbalance between the body oxidants and antioxidants in favor of the former [5] and has been widely studied in association to ultra-endurance sports. However, although it is clearly demonstrated the increase in reactive oxygen species (ROS) and free radicals, the effects of these extreme endurance exercises on the cellular antioxidant defense system, and the oxidative damage to macromolecules, remain to be fully clarified [6]. In this regard, an increase in different plasmatic antioxidant compounds such as glutathione (GSH), thioredoxin or paraoxonse, as well as in the total antioxidant capacity [7,8,9,10], supports the hypothesis of a compensatory mechanism to counteract the increased oxidative stress elicited by the intense exercise in these runners. Conversely, other researchers also showed contradictory and inconclusive results regarding the effects of intense activity on the antioxidant system. At this respect, several studies showed that the antioxidant system remained unchanged or even a decrease in several antioxidant molecules such as glutathione, catalase, or superoxide dismutase [11,12,13,14].

In this line of evidence, similar inconclusive results have been found regarding ultra-endurance activity effects on oxidative damage to macromolecules such as proteins, deoxyribonucleic acid (DNA) or lipids [15,16]. It has been demonstrated that the optimal muscle contractile function depends on the cellular redox state. However, the effects of ROS as well as several antioxidant compounds on contractile function during fatigue and recovery are still being debated [17,18].

Given this, the purpose of this study was to elucidate the impact of ultratrail endurance exercise on several plasma oxidative stress markers with especial focus on the post-race recovery. The objectives of this study are:

i. To determine four plasma oxidative stress markers of long-distance amateur runners throughout the study. Two of them are macromolecule oxidative damage markers (MDA and CG) and the other two are antioxidant enzymes (GR and GPx).
ii. To evaluate the influence of physical variables (sex and age) on these plasma oxidative stress markers after the race, 24 and 48 h afterwards.
iii. To study the possible correlation between baseline values of these plasma oxidative stress markers and the post-race degree of systemic inflammatory processes, loss of skeletal muscle strength and muscle membrane disruption.

## 2. Materials and Methods

### 2.1. Participants

Forty-seven recreational ultra-endurance athletes (29 males and 18 females) were recruited to participate in the study that was developed at the Penyagolosa Trails CSP race in 2019. The track consisted of 107.4 km, starting at an altitude of 40 m and finishing at 1280 m above the sea level, with a total positive and negative elevation of 5604 and 4356 m respectively. All volunteers were fully informed of the procedure and gave their written consent to participate. They were also allowed to withdraw from the study at will. A questionnaire was used to collect demographic information, the history of training and competition and the consumption of antioxidant supplements in the preparation of the race and in the development of the same. The investigation was conducted according to the Declaration of Helsinki and approval for the project was obtained from the research Ethics Committee of the University Jaume I of Castellon (Expedient Number CD/007/2019). This study is enrolled in the ClinicalTrails.gov database, with the code number NCT03990259.

In order to have homogeneous groups, the participants were grouped by age as follows; under 38, between 38 and 45, and over 45 years.

### 2.2. Blood Sampling and Analysis

Blood samples were collected from an antecubital vein by venipuncture at the time of race number collection which was 8 to 6 h before the start, after crossing the finishing line, 24 and 48 h post-race using BD Vacutainer PST II tubes. Samples were centrifuged at 3500 rpm for ten minutes and kept at 4 °C during transport to Vithas Rey Don Jaime Hospital (Castellon), where they were processed using the modular platform Roche/Hitachi clinical chemistry analyzer Cobas c311 (Roche Diagnostics, Penzberg, Germany), as previously published [1,19]. Lactate dehydrogenase (LDH) and creatin kinase (CK) were used to assess muscle membrane disruption, as a surrogate for muscle damage. C-reactive protein (CRP) as an indicator of acute inflammatory reaction [3] (supplementary Table S1).

The oxidative stress biomarkers used in the present investigation were GR, GPx, MDA and CG, which were analyzed as follows:

GPx activity, which catalyzes the oxidation by H_2_O_2_ of glutathione (GSH)to its disulfide (GSSG), was assayed spectrophotometrically as reported by Lawrence et al. [20] toward hydrogen peroxide, by monitoring the oxidation of nicotinamide adenine dinucleotide phosphate (NADPH) at 340 nm. The reaction mixture consisted of 240 mU/mL of GSH disulfide reductase, 1 mM GSH, 0.15 mM (NADPH) in 0.1 M potassium phosphate buffer, pH 7.0, containing 1 mM ethylethylenediaminetetraacetic acid (EDTA) and 1m sodiumazide; a 50 μL sample was added to this mixture and allowed to equilibrate at 37 °C for 3 min. Reaction was started by the addition of hydrogen peroxide to adjust the final volume of the assay mixture to 1 mL.

GR activity was determined spectrophotometrically using Smith proposed method [21]. Briefly, when the GR catalyzed reduction of GSSG to GSH is produced in presence of 5,5′-dithiobis (2-nitrobenzoic acid) (DTNB), 2-nitrobenzoic acid is formed as a subproduct, which formation is monitored at 412 nm. The GSSG reduction was started by adding 25 µL of brain sample to a solution containing DTNB 3 mM prepared in 10 mM phosphate buffer, 2 mM NADPH, 10 mM MEDTA in 0.2 M pH 7.5 phosphate buffer.

MDA concentration was measured by liquid chromatography according to a modification of the method of Richard and coworkers [22], as previously reported [23]. Briefly, 0.1 mL of sample (or standard solutions prepared daily from 1,1,3,3-tetramethoxypropane) and 0.75 mL of working solution (thiobarbituric acid 0.37% and perchloric acid 6.4%; 2:1, *v/v*) were mixed and heated to 95 °C for 1 h. After cooling (10 min in ice water bath), the flocculent precipitate was removed by centrifugation at 3200× *g* for 10 min. The supernatant was neutralized and filtered (0.22 µm) prior to injection on an ODS 5 µm column (250 × 4.6 mm). Mobile phase consisted in 50 mM phosphate buffer (pH 6.0): methanol (58:42, *v/v*). Isocratic separation was performed with 1.0 mL/min flow and detection at 532 nm.

CG were determined to evaluate protein oxidation in milk samples. The CGs released during incubation with 2,4-dinitrophenylhydrazine were measured using the method reported by Levine et al. (1990) [24] with some modifications introduced by Tiana et al. (1998) [25]. Briefly, the samples were centrifuged at 13,000× *g* for 10 min. Then, 20 mL of brain homogenate was placed in a 1.5 mL Eppendorf tube, and 400 mL of 10 mM 2,4 dinitrophenylhydrazine/2.5 M hydrochloric acid (HCl) and 400 mL of 2.5 M HCl were added. This mixture was incubated for 1 h at room temperature. Protein precipitation was performed using 1 mL of 100% of TCA, washed twice with ethanol/ethyl acetate (1/1, *v/v*) and centrifuged at 12,600× *g* for 3 min. Finally, 1.5 mL of 6 N guanidine, pH 2.3, was added, and the samples were incubated in a 37 °C water bath for 30 min and were centrifuged at 12,600× *g* for 3 min. The carbonyl content was calculated from peak absorption (373 nm) using an absorption coefficient of 22,000 M^−1^cm^−1^ and was expressed as nmol/mg protein.

Biochemical results obtained immediately post-race were adjusted by employing the Dill and Costill method [26], using hematocrit and hemoglobin to determine the magnitude of plasma volume changes after the race in each participant.

### 2.3. Muscle Strength. Squat Jump (SJ) and Handgrip (HG) Strength Assessment

The SJ is a validated research test based on three parameters (body mass, jump height and push distance), which allows to accurately assess the strength, speed and power developed by the extensor muscles of the lower extremities during squat jumps [27].

Grip strength, short-term maximal voluntary force of the forearm muscles, measured by dynamometry, is well established as an indicator of muscle status [28]. Grip strength provides a direct measure of the hand skeletal muscle strength. It has been described as an strength index, endurance and general muscular status because its association between peripheral strength and exercise capacity [29].

Previous studies have also suggested that the strength decline index (SDI), calculated as the decline in strength as a proportion of baseline values, measured through tests such as the HG and SJ, is a useful assessment of muscle fatigue [30].

Volunteers were familiarized with procedures concerning strength assessment during an informative session prior to the investigation. HG and SJ tests were performed before the race and within 15 min after the race. In the HG assessment, volunteers remained in standing position, arm by their side with full elbow extension, holding the grip dynamometer (T.K.K. 5401 GRIP-D, Takei Scientific Instruments Co., Tokyo, Japan) in their dominant hand. They were asked to squeeze the dynamometer for 5 s and the test was performed twice, with 30 s of rest in between attempts. Each individual’s peak value was retained for statistical analysis. Following previous studies [31,32], pre to post-race change in HG, given that upper-limb muscles could be considered as being hardly no-exercising muscles during the race. In the SJ assessment, participants were asked to jump as high as possible from a starting position with hips and knees flexed 80 degrees and hands stabilized on hips to avoid arm-swing. Jump height was estimated by the flight time measured with a contact platform (Chronojump, Barcelona, Spain). The test was performed twice, with 90 s of rest in between attempts. Each individual’s best performance was retained for statistical analysis [3] (Supplementary Table S1).

### 2.4. Basal Metabolic Rate (BMR), Body Mass Index (BMI), and Body Composition Assesment

Volunteers height and weight were measured before the start the day of the race. Participants were also subjected to a body composition evaluation test (Tanita BC-780MA, Tanita Corp., Tokyo, Japan). The BMR is defined as the daily rate of energy metabolism an individual needs to sustain in order to preserve the integrity of vital functions. The BMR formula (BMR = Kg × 1 Kcal/h) was calculated based on previous studies [33].

### 2.5. Statistical Analysis

Statistical analyses were carried out using the Statistical Package for the Social Sciences software (IBM SPSS Statistics for Windows, version 25.0, IBM Corp., Armonk, NY). Normal distribution of the variables was verified through the Shapiro-Wilk test (*p* > 0.05) [34].

A Pearson or Rho Spearman correlation analysis was used to assess whether the concentration of oxidative stress biomarkers (GR, GPX, MDA and CG) was interrelated or related to the loss of upper (HG) and lower limb (SJ) strength, hematologic variables of systemic inflammation (CRP) and muscle damage (CK, LDH), as well as cardiopulmonary exercise test results. Post-race and at 24 and 48 h values for these variables (GR, GPX, MDA, CG, HG, SJ, CRP, CK and LDH) for each participant were related to the individual pre-race level to define the delta scores (Δ): Δ (fold increase) = (post-race value − Pre-race value)/Pre-race value [3].

On the other hand, the quantitative variables of oxidative stress were compared using the Student method Tests or U Mann Whitney in each of the sectors where measurements were taken (pre-race, finish line, 24 and 48 h after the race) when they existed two categories and ANOVA test or Kruskall Wallis when there were more categories. Post-hoc comparisons were performed using Bonferroni adjustment for multiple comparisons.

The meaningfulness of the outcomes was estimated through the partial estimated effect size (η2 partial) for ANOVA and Cohen’s d effect size for pair wise comparisons. In the latter case, a Cohen’s d < 0.5 was considered small; between 0.5–0.8, moderate; and greater than 0.8, large [35]. Likewise, correlations > 0.5 were considered strong, 0.3–0.5, moderate and <0.3, small. The significance level was set at *p*-value < 0.05 and data are presented as means and standard error of the means (±SEM).

Finally, the multiple regression analysis was performed using the forward stepwise method. Only normally distributed variables were used as dependent variables. Among the different models obtained, the parsimony principle was applied [36]. Given our limited sample size and the non-normal distribution of independent variables, residual errors from the resulting models were inspected to ensure their normal distribution and thus the reliability of our regression models [37]. To identify the predictive value of the model, the Cohen criterion [38] was applied to one-way ANOVA models. This criterion indicates that R^2^ values less than 0.10 do not present a relevant explanatory value; an R^2^ between 0.10 and 0.25 indicates a dependency of the analyzed variables variance explanation for the identified factors; and R^2^ values above 0.25 is possible to affirm that the explanatory model clinically relevant.

## 3. Results

### 3.1. Demographic Characteristics of The Participants

Thirty-two runners reached the finish line. Nineteen were male and thirteen females, with an average finish time of 21 h 21 min ± 3 h 28 min. All levels of performance were represented in our sample, as shown by their rank, ranging from 7th to 32nd. The main characteristics of these runners are described in Table 1, including sex and age differences. As expected, males showed significant higher pre-race values in weight, BMR, BMI and percentage of muscular mass when compared with female runners. No differences were found in training characteristics or experience between male and female runners. The runner’s age did not affect any of the parameters measured except for the weekly running volume that was smaller in the senior runner group.

### 3.2. Analysis of Plasma Markers of Oxidative Stress

Descriptive data of oxidative stress biomarkers pre-race (baseline), finish line and after 24 and 48 h post-race are depicted in Table 2. Regarding the antioxidant defenses, no significant changes were observed in GPx activity. The GR activity was significantly enhanced in the finish line. The GR enzymatic activity reached the highest value 24 h post-race and returned to normal values after 48 h. Lipid peroxidation (MDA concentration) was also increased in the finish line, declined 24 h post-race, and was significantly increased after 48 h. Oxidative damage to proteins (CG content) also increased immediately after the race and remained elevated 48 h later.

### 3.3. Influence of Sex and Age in the Plasma Markers of Oxidative Stress Evolution of the Runners

**Figure 1:**
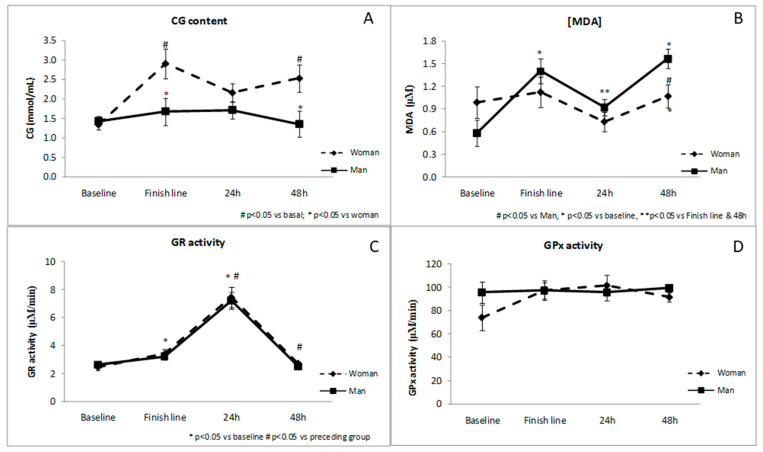
Changes in plasma markers of oxidative status throughout the study according to runners’ gender. CG (carbonyl groups) content (**A**), MDA (malondialdehyde) (**B**), GR (glutathione reductase) activity (**C**) and GPx (glutathione peroxidase) activity (**D**).

Data depicted in Figure 1, demonstrate that female runners have a significantly higher CG content when compared to males at the end of the race and 48 h later. To the contrary, MDA concentration 48 h post-race is higher in male compared to female runners. The antioxidant defenses, measured as GR and GPx enzymatic activity, are not conditioned by runner’s sex.

**Figure 2:**
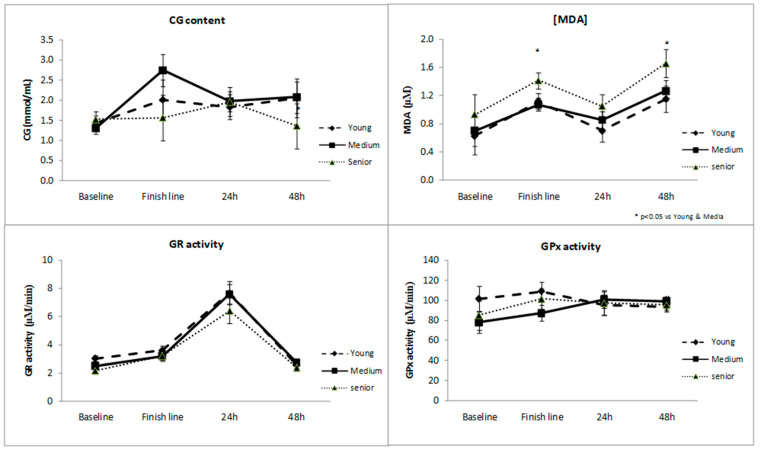
Changes in plasma markers of oxidative status throughout the study according to runners’ gender. CG (carbonyl groups) content (**A**), MDA (malondialdehyde) (**B**), GR (glutathione reductase) activity (**C**) and GPx (glutathione peroxidase) activity (**D**).

Data from Figure 2 shows the effect of the runner’s age. A tendency can be observed in the plasma oxidative status of the senior runners which appears to be globally worse compared to the in the younger runner’s values. Briefly, less antioxidant defenses and higher levels of oxidative damage to lipids and proteins. However, only the lipid peroxidation (MDA concentration) was significantly affected by the age. Thus, the MDA concentration was significantly higher in the senior runners when compared to the middle age and young competitors at the finish line and 48 h post-race.

### 3.4. Correlation between the Plasma Markers of Oxidative Stress and the Skeletal Muscle Force Production, Muscle Damage and Systemic Inflammatory Response

Regarding the post-race skeletal muscle strength, Table 3 shows that there was significant negative correlation between basal MDA concentration and the fold increase in HG values in the finish line. We also observed a significant positive correlation between basal GR activity and the fold increase in SJ values at the finish line.

In addition, basal GR activity negatively correlated with the delta values of LDH at the finish line, 24 h and 48 h post-race. Moreover, basal GR activity negatively correlated with the fold increase of CK at the finish line. It was also demonstrated a significant positive correlation between basal CG content (oxidative damage to proteins) and the rise in LDH observed 24 h and 48 h post-race (Table 3). The inflammatory response observed after the race did not correlate significantly with any of the plasma markers of oxidative stress parameters measured.

### 3.5. Multiple Regression Analysis

Results of the multiple regression analysis are listed in Table 4. When performing the multiple linear regression analysis using Age and GR as the predictive variables, a significant regression equation was obtained for the SJ delta Value dependent variable. This analysis would indicate that younger age as main predictor and higher basal GR concentration, a lower SJ delta value after finishing the ultramarathon was obtained. This regression model predicts the 28.3% of the variance. Another multiple linear regression model was obtained in which the dependent variable was the Δ CK finish line value and the main predictive variables were Age and GR. In this scenario, the regression analysis model predicts a 18.3% of the variance.

As we have previously mentioned, according to the criterion proposed by Cohen [38], our regression models might be considered for their predictive value, explaining within a multicausal model context the influence of oxidative stress on muscle damage and fatigue after a severe effort such as running an ultramarathon.

## 4. Discussion

The present investigation aimed to ascertain the relationship between several athlete’s plasma markers of oxidative stress and the degree of muscle strength and damage after ultraendurance exercise. Results of this study were also extended to investigate the possible runner’s sex and age influence. It is important to remark that this short of studies present limitations regarding the sample size due to the difficulty to finish the competition by the runners. The demographic/anthropometric characteristics of the present study (age, body composition and resistance training) as well as muscle damage, acute inflammation and muscle strength variables have been previously analyzed and discussed by our group [3] (See Supplementary Table S1).

Briefly, male participants showed significant higher pre-race values for weight, BMR, BMI and percentage of muscular mass when compared to women runners [31,32]. The muscular membrane disruption variables, LDH and CK release, peaked after the race and returned to normal values after 24 h. In the Supplementary material section, we also have included results about acute inflammatory processes (CRP), muscle damage (CK and LDH) and muscle strength (SJ and HG), previously published by our research group [4]. No changes were observed in the performance of SJ and HG before the race when compared with the finish line. Both acute inflammation and muscle damage were observed in the finish line, as well as 24 h and 48 h post race.

Regarding the plasma markers of oxidative stress, a time-course analysis of GPx and GR activity, and the oxidative damage to lipids and proteins (MDA and CG, respectively) was performed at the finish line, 24 h and 48 h post-race. It has been showed that ultra endurance exercise is associated with a notably enhanced rate of oxygen utilization and the generation and accumulation of ROS [6]. Moreover, the glutathione system increases its activity to restore the cell redox balance when the formation of ROS is enhanced. Our data showed an increase of the oxidative damage to macromolecules (lipids and proteins) indicating an increase of ROS cellular levels. Thus, lipid peroxidation damage appears to be increased in the finish line and 48 h post-race as confirmed by the MDA levels, proving the presence of oxidative damage to lipids two days after the extreme exercise. A partially recovering effect was observed 24 h after the race as can be seen in Table 2. At this respect, previous studies have shown controversial results regarding the blood MDA levels after ultraendurance exercise. Several studies showed an increase on the cellular MDA levels [14,16,39] compared to data supporting no lipid peroxidation effect after extreme endurance exercise [12,13]. In addition, Skenderi et al., [9] demonstrated an MDA levels decrease 48 h post-race when compared to control and post-race values. However, in this study, the sport type (running, swimming), the distance, the accumulated altitude or the anthropometric characteristics of the sample might be influencing these results. It is also noteworthy to mention that these differences can be attributed to methodological aspects. Authors used the TBARS (thiobarbituric acid-reacting substances) technique which, appear to be a less robust measure of lipid peroxidation [6].

The analysis of the CG content remained significantly elevated for all the time points of the study (finish line, 24 h post-race and 48 h post-race). Interestingly, Spanidis et al., [12] did not find significative differences for this parameter after an ultramarathon mountain race. This discrepancy is explained due to a sample size effect. In contrast, Turner et al., [16] demonstrated an increase in plasma CG content after an ultramarathon race, thus confirming oxidative damage to proteins after ultraendurance exercise.

Regarding the glutathione system enzymes, no significant differences were observed for the GPx activity. Conversely, the GR activity showed a significant increase in the finish line that was even greater 24 h post race, interestingly concurring with the partial MDA concentration recovery. Although both enzymes are mainly located in the intracellular compartment, their plasmatic activity have been broadly used as a measurement of the antioxidant status [40,41,42,43]. In the case of ultraendurance exercise, previous studies show contradictory results to what concern to the antioxidant enzymes [7,12,14,39], but our results support the hypothesis of a compensatory mechanism based on a temporary increase of the antioxidant defense to compensate an oxidative insult [44,45]. Although the activity of these two enzymes has been used to evaluate the presence of oxidative stress, GR is considered the limiting factor of these antioxidative system [46]. It is plausible that the increase of GR activity is not accompanied by an enhanced GPx activity because of other antioxidant enzymes such as catalase or paraoxonase also able to degrade hydrogen peroxide that could be affected by the intense exercise [10,47].

Moreover, it is remarkable that we report a significant negative correlation between the basal GR activity of the runners and the degree of muscle membrane disruption after the race. Thus, the basal activity of this enzyme correlated with [LDH] in the finish line, after 24 h and 48 h and with the [CK] in the finish line and after 24 h. In addition, the resting levels of oxidative damage to proteins (CG content) also showed a significant positive correlation with the magnitude of post-race muscle injury ([LDH] after 24 h and 48 h). It is important to notice that we assume that serum CK and LDH assess for muscle membrane disruption and do not necessarily correlate with muscle structural damage. Furthermore, we have also reported a significant positive correlation between basal GR activity and the improvement in the SJ performance as well as, a significant negative correlation between the basal levels of lipid peroxidation (MDA concentration) and the enhancement in the HG execution. Although there is a limitation on the correlation’s coefficient power to assume an evident causality between oxidative stress and muscle fatigue, these novel findings, suggest that a stronger basal plasma oxidative status might improve muscle strength during ultraendurance sports practice. However, further studies are necessary to increase the number of research volunteers and validate the present results.

Finally, we have reported sex differences in oxidative damage to macromolecules. Surprisingly, female athletes showed higher CG content and less MDA levels than male athletes, although female athletes have less muscular mass and higher body fat mass percentage. There is almost no literature considering sex differences in type of events, probably due to the difficulty in getting sufficient sample sizes. Although several studies include female runners in their research, they do not describe sex differences in the parameters measured [48,49]. Recently Devrim-Lanpir et al., reported significant interaction between time at exhaustion and dietary antioxidant intake in males, but not in females, who underwent an acute exhaustive exercise test (a cycle ergometer) followed by a treadmill test in a laboratory [50]. Moreover, the results of the multivariate analysis show us the predictive value of basal GR concentration and sex in relation to muscle fatigue and cell damage, after ultramarathon.

## 5. Conclusions

Interestingly, the study yielded new results regarding the age of the runners. Senior runners (45–53) showed significant higher levels of lipid peroxidation (MDA concentration) than medium (38–44) and young runners (31–37) throughout the study. A plausible explanation would be the higher body fat percentage observed in senior runners, together with the loss of muscular mass compared to younger runners (Table 1). These results are in agreement with Hattori and cols. who reported that ultramarathon runners aging less than 45 years old had lower ROS levels at all race points [51]. Again, larger series would be necessary to study the predictive value of this assay to consider if a personalized antioxidant supplementation might promote the physiological recovery after great physical efforts.

## Figures and Tables

**Table 1:** Baseline characteristics of the runners which completed the race by sex and age (Average ± SE).

	Total (*n* = 32)	Males (*n* = 19)	Females (*n* = 13)	Young (*n* = 10)	Medium (*n* = 14)	Senior (*n* = 8)
Age (years)	40.9 ± 1.0	40.1 ± 1.2	42.2 ± 1.7	34.6 ± 0.6	41.4 ± 0.5	48 ± 1.1
Weight Pre-race (Kg)	66.1 ± 1.9	73.2 ± 1.5	55.7 ± 1.4 *	68.4 ± 2.9	63.1 ± 2.3	68.4 ± 5.1
BMR Pre-race (Kcal)	1619 ± 52	1835 ± 30	1302 ± 32 *	1708 ± 84	1563 ± 69	1604 ± 135
BMI Pre-race (kg/m^2^)	22.9 ± 0.4	23.8 ± 0.4	21.7 ± 0.6 *	23.2 ± 0.4	22.25 ± 0.5	23.8 ± 1.1
% Body Fatty Pre-race (%)	15.9 ± 1.1	12.4 ± 0.8	20.9 ± 1.2 *	14.3 ± 1.8	14.9 ± 1.2	19.5 ± 2.7
% Muscular Mass Pre-race (%)	84.1 ± 1.1	87.6 ± 0.8	79.1 ± 1.3 *	85.7 ± 1.8	85.1 ± 1.2	80.5 ± 2.7
Number of years running	8.0 ± 0.5	8.0 ± 0.6	8.1 ± 0.9	7.7 ± 1.0	7.7 ± 0.9	9.0 ± 1.0
Number of races > 100 km	2.5 ± 3.3	3.0 ± 0.6	2.0 ± 1.1	2 ± 1.1	2.4 ± 1.0	3.1 ± 0.5
Weekly training days	4.8 ± 1.2	4.7 ± 0.3	4.8 ± 0.3	4.6 ± 0.3	5.2 ± 0.3	4.3 ± 1.0
Weekly running volume (km)	70 ± 22	71 ± 5.8	74 ± 3.7	79.3 ± 8.4	73.4 ± 5.1	53.3 ± 4.5 ^#^
Weekly positive elevation (m)	1771 ± 691	1869 ± 175	1631 ± 157	1600 ± 175	2057 ± 154	1488 ± 318
Weekly training hours	9.6 ± 4.2	10 ± 0.9	9 ± 1.3	10 ± 1.2	9.9 ± 1.2	8.6 ± 1.6

Data partially published previously by our group (Martinez-Navarro et al., 2020) [3]. Abbreviations: BMR: Basal Metabolic Rate; BMI: Body Mass Index. * *p* < 0.05 vs. Males; ^#^ *p* < 0.05 vs. Young and Medium.

**Table 2:** Changes in plasma markers of oxidative stress throughout the study period (Average ± SE).

	Baseline	Finish Line	24 H Post-Race	48 H Post-Race
GPx (µmol/L × min)	87 ± 7	97 ± 5	98 ± 6	96 ± 3
GR (UI/mL)	2.6 ± 0.1	3.3 ± 0.2 ^#^	7.3 ± 0.5 *^#^	2.6 ± 0.1 *
MDA (µM)	0.8 ± 0.1	1.3 ± 0.1 ^#^	0.9 ± 0.1 *	1.4 ± 0.1 *^#^
CG (nmol/mL)	1.4 ± 0.1	2.2 ± 0.2 ^#^	1.9 ± 0.2 ^#^	2.1 ± 0.3 ^#^

* *p* < 0.05 vs. preceding time point; # *p* < 0.05 vs. baseline value.

**Table 3:** Significant correlations between baseline plasma markers of oxidative stress and Delta values of muscle strength (SJ and HG) and muscle damage (CK and LDH).

	R Value	*p* Value
GR (UI/mL)/Δ SJ Finish line	0.405	0.027
GR (UI/mL)/Δ CK Finish line	−0.411	0.019
GR (UI/mL)/Δ ck 24/GR (UI/mL)	−0.352	0.048
GR (UI/mL)/Δ LDH Finish line	−0.402	0.023
GR (UI/mL)/Δ LDH 24	−0.418	0.017
GR (UI/mL)/Δ LDH	−0.406	0.021
GPx (µmol/L × min)/Δ CK 48	−0.360	0.043
CG (nmol/mL)/Δ LDH 24	0.358	0.048
CG (nmol/mL)/Δ LDH 48	0.363	0.045
MDA (µM)/Δ HG Finish line	−0.379	0.032

**Table 4:** Linear regression models.

Model	R^2^ Adjusted	Standardized Coefficients Beta	Standard Error	F (*p*)
Dependent Variable: Δ SJ finish line Covariables: Age, GR.	0.283	0.217	0.16389	0.6521 (0.005)
Dependent Variable: Δ CK finish line Covariables: Age, GR.	0.183	−0.413	0.15558	3.431 (0.002)

Abbreviations: Δ SJ (Fold increase Squat Jump); Δ CK (Fold increase Creatine Kinase); GR (Glutathione reductase).

## Data Availability

Data is contained within the article or supplementary material.

## Supplementary Materials

The following are available online at https://www.mdpi.com/2076-3921/10/3/355/s1, Supplementary Table S1. Evolution of muscle strength (SJ and HG), muscle damage (LDH and CK) and acute inflammation (CRP) biomarkers.
