# Supplementary material for: Impact of Plasma Oxidative Stress Markers on Post-race Recovery in Ultramarathon Runners: A Sex and Age Perspective Overview
Source: Antioxidants (Basel). 2021 Feb 27;10(3):355. doi: 10.3390/antiox10030355 (PMC7996940; doi:10.3390/antiox10030355)
Supplement: Supplementary file 1 [file antioxidants-10-00355-s001.pdf]

Supplementary Table 1. Evolution of muscle strength (SJ and HG), muscle damage (LDH and CK) and acute inflammation (CRP) biomarkers (Average ± SE).

	BASELINE	FINISH LINE	24H POST-RACE	48H POST-RACE
**SJ (CM)**	23.9 ± 0.8	18.4 ± 0.7		
**HG (KG)**	41.6 ± 1.8	41.0 ± 2.1		
**CK (UI/L)**	203 ± 39	4915 ± 709#	2687 ± 431*#	1438 ± 294*#
**LDH (UI/L)**	189 ± 5.5	373 ± 20#	322 ± 20#	312 ± 22#
**CRP (mg/Dl)**	0.16 ± 0.08	2.0 ± 0.2#	3.8 ± 0.4*#	1.9 ± 0.2*#

Data previously published by our group (Martinez-Navarro et al., 2020) [3]. * $p<0.05$ vs. preceding time point; # $p<0.05$ vs. baseline value
